# Communication Adjustment in Engineering Professional and Student Project Meetings

**DOI:** 10.3390/bs10070111

**Published:** 2020-07-06

**Authors:** Kristina Nestsiarovich, Dirk Pons, Sid Becker

**Affiliations:** Department of Mechanical Engineering, University of Canterbury; 20 Kirkwood Ave, Christchurch 8041, New Zealand; kristina.nestsiarovich@pg.canterbury.ac.nz (K.N.); sid.becker@canterbury.ac.nz (S.B.)

**Keywords:** organisational development, team roles, project communication, communication adjustment

## Abstract

Background: communication is important for project teams. There is a need to better understand how members respond to communication at project meetings, and how this affects the team roles the participants adopt. Methods: observational data were collected from (a) two engineering organisations and (b) five university engineering student teams. A mixed methods approach was used, comprising observations (recorded with the interaction diagram method), questionnaires and interviews. Results: participants adjusted their communication style to the behaviour of other people and to different communication settings. This happened with three different dynamics: micro-level (grounding processes in conversation), mezzo-level (emotional and rational regulation) and macro-level (over a period of time). Originality: a new theory was presented for the process of team behaviour during project meetings; specifically, role adoption and communication behavioural changes. Participants change their team roles within three different dynamics: at the macro-, mezzo- and micro-levels, corresponding to the organisation, project and meeting, respectively. The changing of team roles in project meetings arises from rational and emotional regulation. The findings have the potential to assist managers and supervisors to better understand and manage the team dynamics on their projects.

## 1. Introduction

Communication is a key competency for professional engineering practice [1,2]. The importance of this competency is evident in accreditation requirements and is supported by research [2]. Engineers who can convey ideas easily work more effectively and can achieve better results [3,4]. In this context, engineering communication is understood to include: presentations, project discussions at meetings, written communication between engineers or engineering departments such as feedback and emails, vertical communication (between superiors and subordinates), informal communication at the workplace, and non-verbal communication. Engineering communication occurs in the context of people (both professionals and students) engaged in engineering activities, such as design and project management. 

The performance of an engineering team greatly depends on the behaviour of its individual members. To operate effectively, participants should perform their role in the team in a manner that will move the project toward completion. In addition, the nature of the work is meaningful to people at a personal level, affecting motivation; hence, this too needs to be considered. Therefore, there is a need to better understand the roles that project team members adopt and how those communication roles adjust as the project evolves. There is a general tendency in the communication literature to view communication as having two somewhat independent attributes to the interactions: task-oriented and socio-emotional. However, the literature does not robustly show how this is contextualised to engineering activities.

This paper develops a model of the process whereby team members adjust their communication style and team roles to the behaviour of other people and to different communication settings. We show that this happens with three different dynamics: at the micro-level (grounding processes in conversation), the mezzo-level (emotional and rational regulation) and macro-level (the dynamics for the duration of the project or team).

## 2. Literature Review: Project Communication and Team Roles

### 2.1. Project Communication Management

Effective team communication is considered to be a key competence for project managers [5,6,7]. The way this is understood in the standards is primarily around the management of information. For example, ‘Project Communications Management’ comprises “the processes that are required to ensure timely and appropriate planning, collection, creation, distribution, storage, retrieval, management, control, monitoring, and the ultimate disposition of project information” [6]. While there is general agreement in the field regarding the common communication types and communication methods in project management [6], there is a lack of deeper analysis of the communication processes.

The main theoretical framework underpinning the standards is the sender–receiver process (information exchange). This emphasizes the clarity of data conveyance, e.g., the “clarity of messages” [8], and posits that a lack thereof is the cause for misunderstanding. 

The limitation of the ‘sender–receiver’ model is that it portrays the process simply as the mechanical exchange of data, and for this reason has been considered to be a very oversimplified representation of real communication [9,10,11]. The sender–receiver model fails to include the second part of communication: the participants’ interpretations of messages and the creation of shared meaning between people [12]. The idea that each message contains both information and relationship context was expressed in the influential early work of Watzlawick et al. [13]. Although only a premise, rather than an established fact, this type of work did move the field forward. Their applications were primarily related to psychiatric disorders, pathologies, and therapeutic settings, which are less relevant to the present topic. The inclusion of the second part of communication is termed the constitutive approach [14].

Other areas of project communication described in management journals include virtual team communication [15], communication network building [16], conflicts [17], and communication and project performance [18]. In addition, the trust and communication climate of the project group have been considered. For example, it was found that the two key factors in successful team communication are (i) the trust between the task manager and the project coordinator and (ii) the team cohesion [19]. Later, a case study from the construction industry [20] indicated that prior relationships between team members facilitate trust in project teams. 

### 2.2. Social Psychology Literature on Group Development 

Social psychology is rich in the discussion of small team communication and group development. Publications relevant to the present study include Bales [21], Tuckman [22], Gorse and Emmits [23,24], Loosemore [25,26] and others. 

Bales [21] created ‘the equilibrium model of group development’, which explained the development of a team over a period of time. They suggested that there should be a balance (equilibrium) between the task-oriented needs and the socio-emotional needs of the group. Effective teams that are high in cohesiveness and performance can maintain equilibrium between solving tasks and social problems inside the group [27]. However, this balance could be temporary, because the team may meet different situations and move through different stages of progress: the ‘orientation stage’ (group members meet each other, so task-oriented type of behaviour is dominant); the ‘evaluation stage’, when team members actively communicate with each other and exchange opinions; and the ‘control stage’, when team members try to influence the group’s communication. Socio-emotional behaviours increase as the team moves through these stages [28]. Group conflict may occur when a group cannot balance its relational and its task-based interactions [23].

However, other studies in organisational communication [29,30] indicate that communication is less emotional inside the working environment than Bales proposed [31]. Gorse and Emmits [23,24] found that interactions between team members in the construction area were task-based, rather than social-based. Furthermore, online communication has also been reported to have low levels of socio-emotional communication [32]. Hence, the communication medium and environment may predefine the balance between behaviour patterns inside a team [23].

Bales’ model considered only two different categories of team roles: task-oriented and socio-emotional. Communication team roles inside these two big groups were not identified. Another researcher [32] found that the differences in behaviour inside task-based categories were related to the work experience and type of work. It could be interesting to study what else may predefine the distribution of communication interactions inside project teams, and how the engineering context influences the balance between task-oriented and socio-emotional interactions. 

Another communication model was created by Tuckman [22], who proposed the following temporal phases of group development: forming, storming, norming, performing and adjourning. Forming includes orientation, testing and dependence. At these stages, team members are mostly uninformed about project objectives, behave independently and may be focused on themselves. Orientation is conducted through testing—identifying the boundaries of communication between people. Dependence arises as the establishment of relationships between the team leaders and the other team members. Mature members try to model appropriate behaviour. Generally, in this stage of development, the team communication aims to define the scope and the approach of future work [22]. In the Storming stage, resistance to group influence and task requirements may appear, together with conflicts around interpersonal relationships. These problems may be overcome in the third stage, Norming. This is the finding of communication norms through the expression of personal opinions. Finally, the group reaches the fourth stage, Performing, where team roles become flexible and structural problems are solved. This means that the group is ready to deal with the tasks [22]. A fifth stage was added later by Tuckman and Jensen [33], and was called Adjourning, which was characterized by the completion of the task and, finally, the team’s separation.

Brown [34] found that the frequency of communication depends on the phase of the task (and group development, as they are correlated), and that this influences the team’s effectiveness. Teams that meet frequently and discuss the engagement of the participants at early stages generally have better performance than those that do not [35].

In another work [36], the stages of group development were used to study how participants in different groups perceive team communication. Team composition did not correlate with stages of group development, and the perceptions of group interactions were similar among different teams.

A limitation of the above models of group development is that these models only consider group development over the period of the entire project development. They do not include communication change (adjustment) at other levels (during the meeting time or inside the conversation). Hence, there is a need to better understand how behaviour patterns change within a shorter timeframe. 

### 2.3. Team Roles in Project Communication

A team role can be understood as a behaviour pattern; that is, an interaction between the participants of a project team while performing a task [37]. 

#### 2.3.1. Team Roles in Standards

While the standards identify the need for teamwork, e.g., “Teamwork is a critical factor for project success” [6], and the need for awareness of team role models [38], they do not identify the types of roles that might be important. The standards appear to implicitly assume that team roles correspond to technical functions, e.g., “setting out the team roles and critical path for the project” [38]. In contrast, the broader literature on team behaviour presents roles as casual behaviour patterns, i.e., styles of behaviour in different circumstances [39].

Furthermore, there is an expectation that “the individual chooses the appropriate way of communicating” [38], but there is no guidance in the standards on how this choosing process might operate. Furthermore, it is unclear what these different styles of communication are: “The individual chooses the appropriate way of communicating for the target audience. The individual is able to communicate on different level and through different channel” [38].

#### 2.3.2. Team Roles in Management Journals

Many studies have been devoted to the questions of improving communication inside project groups, and to finding correlations between team diversity, performance, leadership and project success [40]. Team roles (a match between team member’s skills, interest and assigned tasks) are considered to be one of the factors that predefine individual communication competency and project success [41,42].

Another work [43] found that there is a strong correlation between team role clarity and individual participants’ satisfaction with the project performance. Team members should know what contribution they are supposed to make in a project’s development.

However, the team roles under consideration were formal (predefined by job duties). There is still a lack of work about informal participant behaviour in project communication, and how these roles may change over time.

#### 2.3.3. Team Roles in Engineering

Communication in the engineering context has been investigated from different perspectives: communication as a technical process, engineering cycle, engineering communication style, communication problems, skills and artefacts. However, the literature is sparse in the specific area of team roles in engineering student teams and in engineering practice. Work in this field is mostly focused on applications in business process re-engineering and construction areas [25,26]. The few relevant studies are summarised below.

The work of Dainty [44] studied communication between project participants from different perspectives, ranging from interpersonal interactions to the organisational level of communication. Team roles taken by participants in construction teams were found to affect their ability to communicate effectively. An incorrect choice of role assignment resulted in communication barriers.

Loosemore [45] found that participants in project construction teams sometimes showed excessively formal behaviour because of contractual procedures, which created anxiety and tension inside the team. He concluded that some level of flexibility is required, especially in crisis situations. In another paper, Loosemore [25] studied the effectiveness of information transfer between project participants, finding weak associations between the centrality of communication structures and the efficiency of communication.

However, it should be noted that the works of Dainty and Loosemore look at communication from sender–receiver perspectives, and do not consider how participants feel in these team roles, how participants perceive information, or how participant behaviour patterns might change during the timeframe of a single meeting. 

#### 2.3.4. Taxonomies of Team Roles

Many different taxonomies of team roles exist in the literature. The most famous among them are Belbin’s [46], Benne and Sheats’ [47], Margerison’s [48], and Parker’s [49].

Belbin’s inventory [46] suggests that the adoption of team roles happens on the basis of personal preferences, whereas Benne and Sheats [47], and Parker [49] focus on extracted personal styles. The Team Management System (TMS) taxonomy was developed by Margerison and McCann [48]. This role inventory is similar to Belbin’s because it is based on the personal preferences of team participants. There are also other taxonomies, e.g., Parker’s taxonomy, that identify four main types of behaviour in teams [49]: contributor, communicator, collaborator and challenger. However, Parker did not call his inventory ‘team roles’, and instead used the term ‘team players’ style’. 

As for team role taxonomies in engineering, the literature is scarce in this area. Platt developed a team role inventory for business process re-engineering [50]. This classification is an adoption of Belbin’s taxonomy for business re-engineering teams. Furthermore, more recently, another work [51] devised a new categorization for team roles designed specifically for engineers in project meeting communication. The roles from this inventory were based on Benne and Sheats’ taxonomy, but were elaborated to suit the needs of engineers (Table 1). Furthermore, later additional roles were added by dividing the Information provider and Connector roles into two subcategories: active and passive. In addition, a circumplex of team roles arranged in a circular order was built to represent different aspects of communication patterns [52].

### 2.4. Gaps in the Body of Knowledge

While the literature identifies that communication styles and team roles change, it primarily addresses the human grounding processes [53,54] and the spoken dialogs system (interactions between human and computer) [55]. It is not specific about the reasons for such change, or the mechanisms.

Furthermore, there are many works devoted to group development that discuss communication problems in small groups. However, there is a lack of literature on the changing of informal team roles in project teams within the engineering context.

The present paper extends the field by identifying how and why the communication adjustment processes occur at the higher levels of team function. 

## 3. Approach 

### 3.1. Research Questions and Methodology 

The primary research questions for this study were ‘How do people adapt to the changing of the communication environment in a project team?’, and ‘Over the lifecycle of an engineering project, how do team roles and communication at project meetings change in time?’. To answer the questions, we investigated two different project group types—(i) engineering student teams at the University of Canterbury, New Zealand, and (ii) engineers in professional practice (New Zealand)—where interactions occur between people as they work on complex projects. 

In this study, multiple qualitative research methods were used: observation, interview and questionnaires. 

Observations of project team meetings occurred on a regular basis: weekly for student teams and daily for commercial organisations. During observations, the researcher sat separate from the group while taking notes using the same ID method as [51]. There was no audio or video recording. A code system was used to record participants’ names. After every meeting, the researcher extracted information from the interaction diagrams to the research journal, and then used NVIVO software (version 12 Pro) for qualitative analysis.

Before the first observation, all participants in the project team meeting received a short questionnaire to provide information about demography, background and social links between each other. This questionnaire was accompanied by a personality test. For this purpose, we selected the IPIP version of the Big Five Markers’ test created by [56], taken from [57]. 

To clarify the behaviour of project team members, we conducted a structured interview with each participant. The participants were interviewed on an individual basis, separately from the observation time. This was done after the last observed meeting. Additionally, each member of one of the student groups was interviewed on two occasions: once in the middle of the academic year and once at the end of the academic year. Data from the interview answers were summarized and compared with observations.

Ethics approval was received from the University of Canterbury Human Ethics Committee (HEC 2017/70/LR-PS), and all participants gave their consent before the study commenced.

The current paper adopted the team role framework of [52]. We selected that inventory because it was designed specifically for engineering team project meetings, and because the team roles described there are informal in nature. 

#### Summary of the ID Method 

The interaction diagram (ID) method of observation [51] was used to capture interactions between the participants of project engineering meetings. This method permits the observation of team communication in a time-pressured situation using only a paper notebook, and therefore without intrusive audio or video recording. This also facilitates ethics approval, and the willingness of commercial firms to agree to the research. In addition, there is much less need for post-event data transcription.

**Figure 1:**
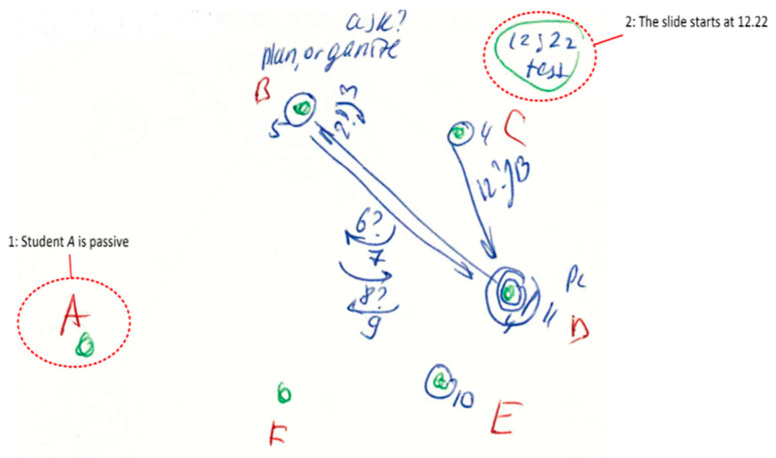
Interaction diagram: communication situation 1 (reproduced from [51] by permission).

In the current study, communication behaviour was recorded by the ID method as a set of written notes and phrases. This helped to identify team roles and the changing of communication patterns during the project communication time. Figure 1 shows an example of this note-taking. 

### 3.2. Information about Teams and Organisations

In the university setting, the communication events of interest were student final year project meetings. These meetings lasted about one hour and happened on a regular basis (weekly). These were large projects that needed the whole academic year for completion. Each team had an engineering problem to solve according to the brief provided by an external client. Students were expected to share details about their project results and to discuss existing problems with team members. Participants generally had one or two official meetings per week, and there could also be other types of communication between them. However, the focus of our research was on the official project meetings of students and supervisor/client that took place at the university or in the external organisation.

Meetings involved four students of similar engineering backgrounds (mechanical or mechatronic) and an academic supervisor (sometimes a client or other guests also visited meetings). A total of five student teams participated, and the study was longitudinal in nature. Students were of the same age group (20–25 years old). There were twenty-one males and four females. 

In the professional engineering setting, the communication events were project design meetings that included engineers of diverse organisational positions. Engineers discussed existing project problems between each other and with the manager of the group, and made plans for future work. Selection criteria were:

- the small size of the team (3–8 members);
- regular team meetings;
- at least one member of the team being from a different engineering discipline;
- at least one participant being from a different official position, e.g., supervisor or manager;
- project discussion being in the initial stage of development (so that the observations did not start midway).

Two project teams from different organisations were followed for their first five meetings.

The first organisation was an engineering consultancy firm situated in New Zealand. The nature of the work was engineering analysis and design in the areas of machines, vehicles and structural engineering. The size of the organisation was fewer than 40 members in total. Projects were short in duration—from one hour to one week. The meetings happened on a daily basis and lasted around 15–20 min. The study followed one project team with six participants. All participants were male engineers, aged between 18 and 25 years (except from one engineer aged 35, with 10 years of experience).

The second organisation was an engineering manufacturing firm situated in New Zealand. The nature of the work was engineering design and manufacturing. The size of the organisation was more than 200 members in total. The firm had many simultaneous projects. The length of a typical project was between three and five years, and projects were divided into several smaller tasks that were assigned to teams of engineers in different discipline areas. The team under observation was a software development engineering team consisting of 11 members (all males, aged from 23 to 55). Most of the participants had many years of work experience in engineering (over 10). However, there was also one recently graduated team member. 

## 4. Results and Findings

### 4.1. Factors That Cause Communication Adjustment

There are many factors that may cause a change in the communication behaviour of participants. To analyse them, we used information from the interview. 

The list of interview questions is provided in the Appendix A. One of the questions explicitly relates to changes in individual behaviour: “Do you feel that you changed your communication behaviour at different meetings? Which communication situations caused that?” Responses from the five student teams and two engineering organisations are summarized below, in Table 2. In doing so, we identified several themes or categories of factors. The primary factor, inasmuch as it was identified by all groups (including supervisors), was Solving Progression issues. The next tier of factors that were common to both student and practicing engineers were Adjustment to Audience and Adopted/assigned roles. Factors that were only identified by practicing engineers were Engagement with Depth of discourse, and Type of meeting. Likewise, factors only identified by student engineers were Defensive behaviour, Growth in personal confidence, and Mood responses.

As evidenced above, the participants of the project teams changed their behaviour in response to different factors. This is a process of communication adjustment.

Given the longitudinal nature of the study, it was possible to observe these interaction dynamics across different timescales. Hence, we propose, based on the observations, that the role adjustments may be categorized into three different timescales or levels: micro-level, being the grounding processes within a conversation cycle; mezzo-level, being emotional and rational regulation during a meeting; and macro-level, being the role dynamics over the duration of the project.

### 4.2. Communication and Behavioural Pattern Change at Macro-Level

At the macro-level, people adjust their behaviour through the changing of communication style and behaviour patterns (team roles) over a long period of time: in the case of the student teams, this was over an academic year. Table 3 shows the change of communication activity of student teams over the project development time.

The results in Table 3 show that the increase of communication activity happened with all teams. A more detailed examination of the changing team roles in student Team 2 was performed, based on interview data in the middle of the academic year and at the end. Students were asked “What is your intuitive perception of your own communication style in this team?” Another question allowed them to identify all of the team roles that they thought described their own behaviour. The list of roles was from [52]. The results are shown in Table 4.

Evidently, the individuals’ intuitive perception of their own communication behaviour did not change over the academic year. Participants described their behaviour with different words, but their self-described main characteristic remained the same. In reality, according to the observation notes, some communication adjustment did occur. This indicated that the individual’s perception of their own behaviour could be different from the observed actual behavioural patterns. 

Thus, participant 2A took more active roles in the team communication as the project developed. They were “taking a larger influence in position over the time due to observing lack of direction/drive in team”. This corresponds to roles of Initiator and Gatekeeper, per the (Nestsiarovich & Pons, 2020) taxonomy.

Participant 2B initially asked many questions; however, over time, they started to provide information, rather than asking. At the end, they also became very passive (Passive Collector) for some reason.

Participant 2C apparently did not notice communication behaviour changes at the meetings. Initially, they were very active (for the first three meetings), trying to facilitate and regulate the communication flow in the group. However, they later became more passive, presumably because of the high communication activity of the other members. 

Participant 2D was passive from the very beginning of the project, and remained mostly passive. However, they later became more sensitive to others’ needs, accepting the role of Gatekeeper. This was probably because, in the second semester, this participant was frequently elected by group members to be a Facilitator (chairing the meeting). 

Finally, participant 2E (the supervisor) was more active in the beginning of the project (Explorer and Information Provider), asking leading questions and providing necessary information. Later, they became more passive (Passive Collector), letting the students initiate and solve minor problems by themselves. 

In general, it seems there was a difference in team behavior in the beginning and at the end of the project. In the beginning, the team reported to the supervisor, rather than discussing problems between each other. By the end of the second semester, the situation changed, with students more actively participating in common discussions. 

### 4.3. Components of Communication Setting

The results show that participants do not automatically use the same communication style during the whole of the project’s duration. Team roles appeared to be very changeable. Roles have been shown to depend on parameters including personal characteristics, the team’s goals, the addition of a new member group and even on the meeting’s location [52]. 

Two components to the communication process were identified. The first is the initial setting of roles (adoption) and the second is the adjustment (the changing of behavior patterns) that occurs during the project’s development. While the macro-level involves the longitudinal change of behaviour across multiple meetings, changes in the participant’s role behaviour are also anticipated at the mezzo- and micro-levels.

The findings suggest that, at the micro-level, two interlocutors change their behavior quickly in response to a situation (e.g., of misunderstanding). The mezzo-level corresponds to the interactions between participants during a single meeting. Hence, the communication dynamics relate to both the organisational scale and the time scale over which people change their behaviour. 

We further propose, based on the observations, that grounding occurs at the micro-level, and regulation at the mezzo-level.

### 4.4. Communication Adjustment at the Micro-Level 

#### 4.4.1. Miscommunication and Non-Understanding

Communication changes at the micro-level were observed to be associated with oral conversation during the project meetings, and these changes were primarily associated with miscommunication, misunderstanding and non-understanding. This is consistent with the literature.

Non-understanding occurs when a person fails to interpret a message at all (not having any hypothesis or ideas) and is aware that it has happened. In contrast, during misunderstanding, a participant believes that his or her interpretation is correct, but this may be far from what the speaker intended [54]. Misunderstanding should not be confused with misconception, which refers to errors in prior knowledge [58]. Generally, non-understanding is recognised immediately, while misunderstanding may not be identified until the conversation is over (or is never identified) [59]. Partial understanding refers to the understanding of some part of the full intention of the other person [59].

#### 4.4.2. Micro Adjustment: Conversational Grounding in Engineering Communication

The micro-level communication adjustment is a reaction to a miscommunication event, whereby interlocutors attempt recovery by regulation or by correction of the communication behaviour. As Clark noted [60], a speaker cannot just deliver message and hope that a listener will understand it. At this level, the adjustment is a grounding process of communication. A typical grounding process involves people giving evidence of understanding or non-understanding [53]. This is evident in non-verbal behaviour, such as facial expressions and gestures, or verbal behaviour, such as posing clarifying questions and asking for information to be repeated. The interlocutor contributes to grounding by seeking information as to whether what they have said has been comprehended. 

**Figure 2:**
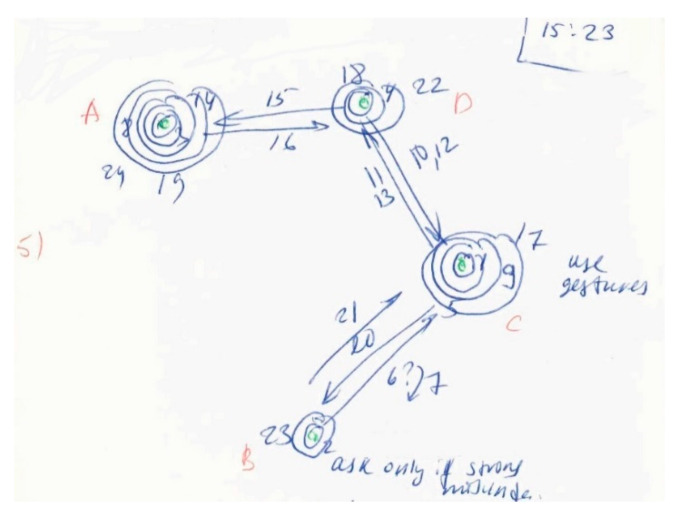
Example of grounding process in a project meeting (participants B and C), represented as an interaction diagram.

During the observations of this study, some micro adjustment processes were also observed. A typical situation we observed for engineering meetings was when a participant made a statement using technical terminology, and the interlocutor misunderstood this statement. We observed the common response was to give a wrong answer, or to ask a clarifying question along the lines of “Sorry, I don’t understand; please repeat”. The first person then stated their matter again, using different words and without special terminology, so that they finally understood each other. We also observed that participants used non-verbal signals, particularly facial expressions and gestures, to show their misunderstanding or to explain something. Observational note-taking in this study used the interaction diagram (ID) method [51]. An example micro adjustment processes is represented in the interaction diagram of Figure 2. 

In Figure 2a, b, c and d are participants in the meeting that communicated between each other. Each interaction (turn-taking) was represented by an arrow, and was assigned a sequence number. Question signs near the number mean questions were asked; arrows above this question sign represent that an answer was given. Circles represent a broadcast addressed to the whole group, rather than to an individual (arrow). As Figure 2 shows, participants B and C misunderstood each other, asking questions and using gestures for explanations. This kind of miscommunication was the most common problem observed in engineering communication at the micro-level.

Another problem was when a participant had insufficient knowledge of a topic and had to ask additional questions to understand. Analysis of the responses to the interview questions showed that these two problems covered most miscommunication events. By asking additional questions, people adapt to the new communication situation, and their team roles may temporally change from Information Provider to Explorer. 

### 4.5. Communication Adjustment at Mezzo-Level

The micro- and macro-behaviour changes interact and occur in parallel during a meeting. Micro adjustments happen regularly, whereas macro regulations were observed less frequently.

#### 4.5.1. Regulation at Mezzo-Level

At the mezzo-level, which corresponds to the entirety of a meeting, the adjustment was observed to have rational and emotional components. Both result in an adjustment to a team role. We propose that the rational component consists of adapting to the procedures and rules of a particular meeting or discussion. This is evident in Table 1 as Solving Progression issues and Engagement with Depth of discourse. 

This is complemented by emotional regulation, where people respond affectively to internal or external stressors [61]. Situations that might elicit emotional responses could be when a person encounters a new environment or communication event, such as a different meeting location or a new member of the team. 

The person feels stress, anxiety, or fear in the situation, which they attempt to control (hence the term ‘regulation’) by internal cognitive processing. This results in temporary mood behaviour such as passivity, change in tone, or neurotic behaviour. A complete emotional adjustment can lead to a return to a normal behaviour, i.e., a return to the team role that is typical for this person. Incomplete emotional adjustment may cause problems such as non-understanding or misunderstanding. If regulation fails, then aggression may occur. Emotional adjustment may be accompanied by non-verbal signs [62].

The emotional regulation factors are visible in Table 1 as Adjustment to Audience, Type of meeting, Defensive behaviour, Growth in personal confidence and Mood responses.

**Figure 3:**
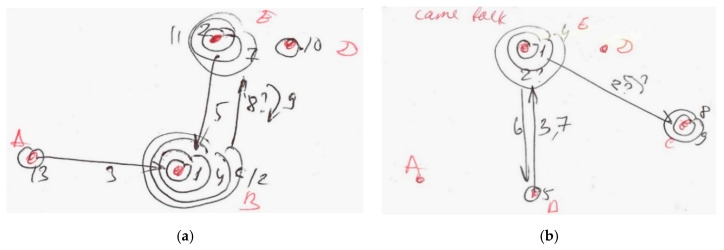
(**a**) Supervisor is absent; (**b**) supervisor (C) came back.

An example from our observations was a new team member who behaved very passively at the first meeting, when joining an already existing group. Newcomers need to determine the behavioural expectations of the team, and to feel comfortable in the new environment. Another important factor was the relationship between participants, particularly the presence or absence of a supervisor or manager in the meeting. Our data showed that, without a supervisor, participants felt more freedom and their behaviour became more natural (in harmony with their character and personal communication preference). An example of such a situation is given in Figure 3a, b below. The team communication intensified without supervision; participant B took a temporary role as Team leader and transmitted information to other group members. Even participant D became more active. When the supervisor returned, the communication within the team was suppressed.

Generalising our observations, we conclude that a typical macro adjustment process involves two key factors. First, it is precipitated by some special situation, such as the arrival or departure of a team member, the introduction of a new participant, etc. Second, the adjustment involves rational and emotional regulation. 

#### 4.5.2. Adjustment by Changing a Team Role 

Our third finding is that another form of adjustment at the mezzo-level is the adjustment of team roles. This happened when one team member suddenly changed their team role (e.g., received a telephone call) or left the meeting. As a result, the other team members adjusted their behaviour, changing to more active or passive roles, sometimes substituting a missing component.

**Figure 4:**
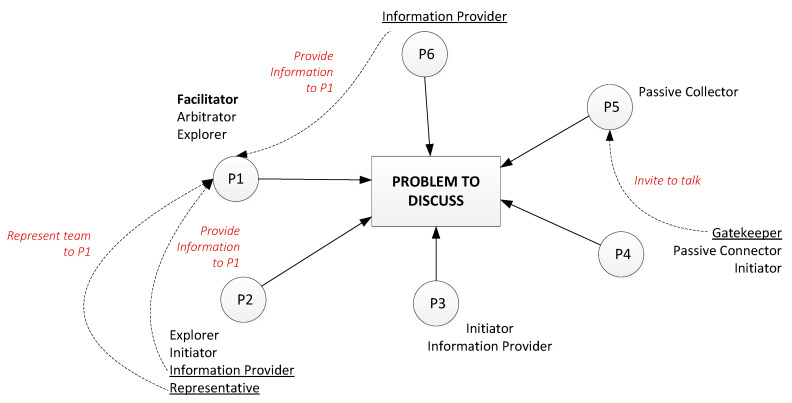
Team role adjustment: stage 1.

**Figure 5:**
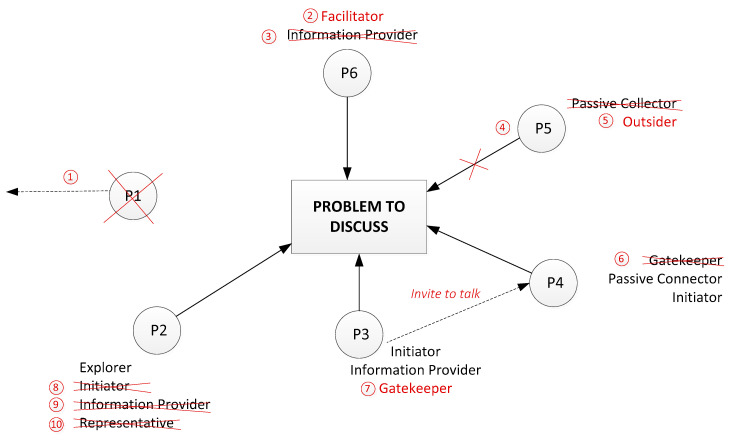
Team role adjustment: stage 2.

Figure 4 and Figure 5 show an example of a team role adjustment that was typical for project student teams. In this example, P1 (participant 1) was a supervisor, participants P2–P5 were students and P6 was a visitor (PhD student). Arrows mean active participation in problem discussion; dashed arrows represent the provision of information to a group member.

The team role changes happened very quickly. Red numbers on Figure 5 show the sequence of events (the changing of team roles). A crossed circle shows the departure of a member; a crossed line indicates active behaviour turned into passive behaviour; a crossed team role (and new roles in red) show changed roles.

Therefore, we propose two stages of team role assignment. The first is a situation prior to the team role change, and the second is the changing of team roles (an adjustment process). An example follows. 

Stage 1: Precursors to Role Change

Stage 1 is an initial communication balance in the student team before supervisor P1 left the room. Initially, P1 covered the role of Facilitator, Arbitrator and Explorer. Participants P2 and P6 provided the supervisor with necessary information (Information Provider). P2 also represented the whole team when supervisor asked a question of them (Representative). Student P3 was the Initiator and Information Provider (they provided information to other participants, not only to supervisor P1). P5 was a Passive Collector, and student P4 tried to involve P5 in more active communication from time to time (Gatekeeper). Furthermore, P4 took the role of Passive Connector and Initiator.

Stage 2: Role Adjustments Occur

After a person with power (P1) left the meeting (event 1), the distribution of team roles changed significantly. Participant P6 took the role of Facilitator instead of the supervisor (event 2), and relinquished the role of Information Provider (event 3). Passive Collector P5 apparently lost interest in the conversation and became an Outsider (out of the communication completely) (events 4 and 5). P4 then stopped attempts to involve P5 in conversation (Gatekeeper), and became more passive too (event 6). P3 took the role of Gatekeeper and invited P4 to talk (event 7). At the same time, P4 continued with their other team roles—Initiator and Information Provider. Finally, student P2 lost their position as Representative and Information Provider because the supervisor left the room, and became less active in communication (events 8–10).

The team role assignments of this group resulted in markedly higher passivity from members after the balance of power in the meeting changed. Apparently, these team role macro adjustments contained both rational and emotional components. The rational component was evident in the creation of new communication procedures and rules to continue discussion without the supervisor. Multiple individual emotional components were evident, as members responded negatively to their peer taking the leadership role. Evidently, participants did not want to continue communication without the supervisor, but were unable to respond rationally. They instead used emotional mechanisms to change to less participatory roles, and hence curtailed the efficacy of the meeting. Altruistic responses were also evident, where individuals helped other members to adapt to the new situation or attempted to involve others in communication. 

Possibly, some of the negative consequences of this event might have been reduced had participant P6 built approval before taking the role of Facilitator, rather than simply capturing it.

The rational and emotional mechanisms for team role adjustment were also evident in other student groups, and in the commercial engineering project meetings.

## 5. Discussion

### 5.1. Model of Communication Adjustment at Micro- and Mezzo-Levels

We propose the following model of the adjustment processes. We use the idea that miscommunication is divided into misunderstanding, non-understanding [54] and partial understanding [59]. We adapted the scheme of error handling from [63].

**Figure 6:**
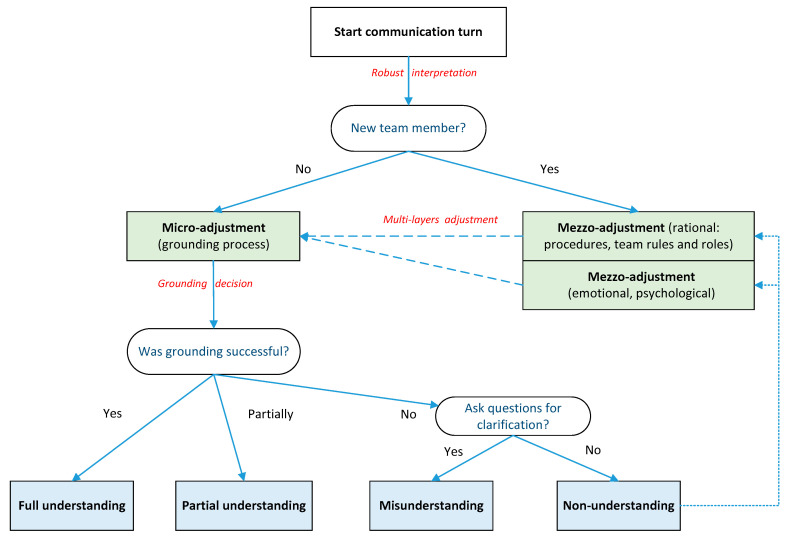
Model of adjustment processes at the micro- and mezzo-level of team communication.

The model assumes a turn-based interaction. First, the speaker starts a new turn in conversation. The listener interprets the speaker’s output, accepting or disagreeing with the speaker’s ideas and attempting to correct contentious areas. If the participant (listener) is not a new member, the normal grounding process starts. The goal is to find common ground by asking questions or showing an utterance of understanding. If the grounding is successful (both members show acknowledgment, confidence and evidence of understanding), then a degree of understanding (full or partial) has been achieved. Otherwise, if participants are still unsure about mutual understanding, then the grounding process has failed. In this case, the outcome is misunderstanding (the distortion of information perception and misinterpretation), or even complete non-understanding. Role changes may occur whereby a participant may abandon the attempt to resolve the communication problem. This process is summarised in Figure 6.

This scheme combines grounding process with macro adjustments, such as emotional regulation and adapting to the rules and procedures of the meeting for the new member. These processes occur simultaneously. A new team member, after passing the basic macro adjustment process, becomes involved in the conversation, along with its possible miscommunication events.

Any team member that fails to understand the team discussion may need to repeat the adjustment process again. This might involve seeking to better understand team goals and rules, obtaining more topic-specific knowledge, and solving any maladapted emotional regulation problems (e.g., changing attitudes to other participants or to one’s own contributions to project completion).

### 5.2. Team Role Adjustment at the Macro-Level of Communication

The above model represents the role adjustment (or grounding) over short time frames, within conversation episodes or within meetings. However, adjustments also occur over the project timeframe, i.e., the macro-level. This is a type of metacommunication, whereby roles change more slowly in response to changes in the technical nature of the work, a change of team membership, or a maturation of the protagonist’s own feelings towards other participants. For example, some observed role adjustments enhanced cooperation and cohesion, while others thwarted the perceived negative behaviours of others, and these responses were deliberate.

Consequently, the long-term development of the team, in terms of the development of cohesive and constructive relationships, is an important factor for role adjustment at the macro-level. We did not specifically look for Tuckman’s temporal demarcations, nor were they obvious in the data in the order he proposed; nonetheless, our observations support the idea that the temporal development of teams involves role adjustments in response to task, social and personal changes, and may be directed towards conflict or cooperation.

According to Bales’ work on group development [21], there should be a balance between the task-oriented and socio-emotional needs of a team. In our case, this could be understood as a balance between team roles that are directed toward task development (to get jobs done) and social development. It is generally accepted that the two dimensions are in conflict, or at least independent of each other, such that an over-emphasis on task completion causes deterioration in group cohesiveness (and the inverse). While this balancing process is commonly anticipated in the literature, it has not been all that clear how it occurs in practice.

**Figure 7:**
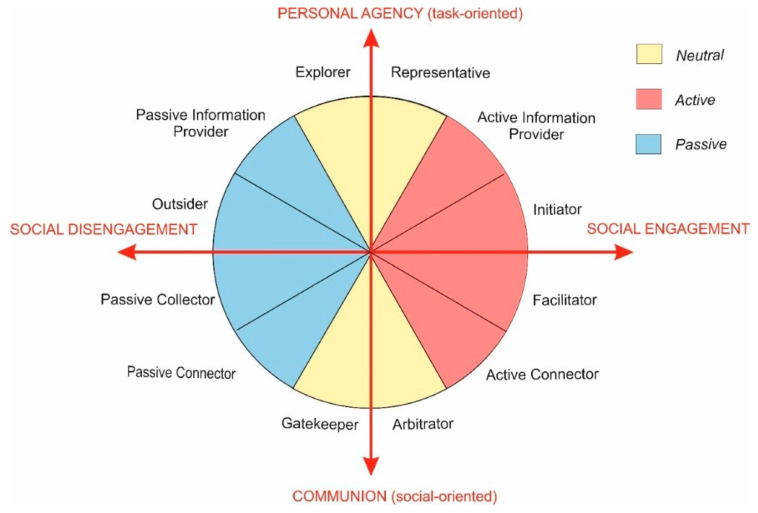
Circumplex of team roles (reproduced from [52] with permission).

We saw evidence for how this balance mechanism occurred in the teams under observation. Participants dynamically adjusted their roles to compensate for what they perceived as shortcomings in the group’s behaviour as a whole. These adjustments occurred very quickly—within minutes—and were primarily characterised by rational or emotional mechanisms, sometimes both. Furthermore, it was evident that participants had a limited range of roles they were able to access in these adjustment situations. There was a stability to the role taken by a member, such that the natural adjustment to a new role was only slightly different to the previous one. This observation implies that there is a progression or scale of roles, and hence it is natural to wonder how that might be arranged. The answer was previously reported by the present authors, based on the same data set [52]. The idea that emerges is of a circumplex of team roles; see Figure 7. This assumes two axes. The first represents a continuum between personal agency and communion. The location of a role on this axis defines the behavioral orientation of a participant toward task completion (to move the project to completion) or solving social problems. For example, the roles of Explorer and Representative are understood in this framework as task-oriented roles, and Gatekeeper or Arbitrator as socially-oriented. 

The second axis shows the extent of involvement in the social team processes, ranging between social engagement and disengagement. For example, Passive Collector and Outsider are passive roles that do not require much interaction with others. 

Thus, the first axis represents the objective—the outcome the participant seeks for the group. The second represents the approach—what engagement style the participant uses. The circumplex represents the idea that adjacent roles are the most accessible locations for role adjustment, and that opposite segments are contrary to each other on the attributes represented by the axes.

**Figure 8:**
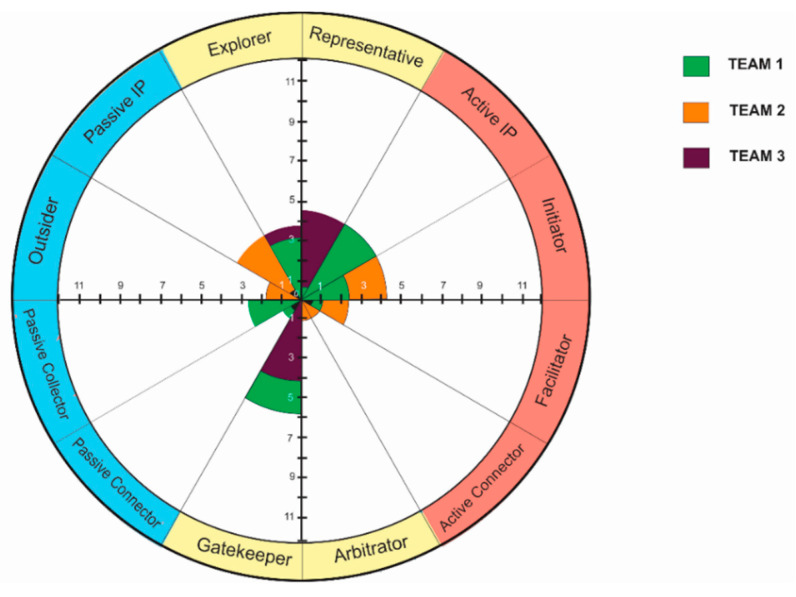
Example of collective role profiles for several teams.

To analyse the balance between roles, a team role profile can be created. See Figure 8 for an example for some of the teams. We refer to this as the collective role profile. 

Numbers on the axes represent how many times this behaviour pattern (team role) was used by team members. This may be performed through observation using the ID method of note-taking, or interview answers. As is evident in the above figure, the teams put the task-oriented roles to greater use than the communion ones, with a tendency to rely on the Gatekeeper role to provide the communion balance.

In our study case with the student teams (Table 4), the collective role profiles did not change to great extent over the duration of observation. Changes tended to be minor. For example, Students B and C became more passive, passing their active social position (the roles of Initiator and Gatekeeper) to students A and D. Supervisor E moved from Explorer (a active task-oriented role) to the more passive role of Passive Connector, giving the opportunity for students to initiate and solve problems by themselves.

It is also important to notice that the taxonomy of team roles describes the casual behaviour of project team members, i.e., according to their personal preferences, rather than the behaviour being defined by official positions. Therefore, keeping a balance between social and task-oriented roles may be hard to regulate for a person with power (a supervisor or manager). 

Our observations showed that, when the project needed a change in the collective role profile, the adjustment was limited to adjacent team roles. It appears that the roles taken by participants were preferred styles of collective interaction, and like personality traits, somewhat fixed, though malleable. Hence, we recommend that, when a participant needs to move to a more active or passive sector of the circumplex, they first try to accept the adjacent team role. For example, a very passive student with the role of Outsider could try to be a little more active at the meeting by taking the role of Passive Collector or Passive Information Provider (Passive IP), thus slowly moving out of their zone of comfort. It appears this may be a promising area for future research, with the potential for significant improvements in human development and mentoring.

### 5.3. Implications for Practitioners

Managers in leadership roles may wish to consider ways of encouraging behaviour that develop team performance and decrease the chance of conflicts or misunderstandings.

At the macro-level, managers could consider creating a trustful environment for the team members so they can feel comfortable communicating in their team roles. Our results show that events that precipitate adverse emotional responses cause members to adjust their roles towards the reduction of the expression of their own ideas. 

Managers might consider managing the team role distribution among participants. People are naturally more comfortable with some roles rather than others [52], but their preferences are not fixed. The present study showed that participants will change roles, and quickly too, as gaps occur in the role structure. From this, we infer that people’s adoption of roles is significantly affected by the non-availability of their preferred roles (perhaps due to other more forceful people taking those roles), and altruistically by their assessment of roles that need filling. Consequently, managers might do more to help develop younger or newer team members by giving them tasks that explicitly require greater communication activity. The opposite is also likely to apply—some team members may have taken roles out of duty, and actually be more comfortable with more passive roles. In this way, the process of communication adjustment could be facilitated by the manager so that members feel more confident about their contributions to the successful completion of the project.

At the mezzo-level, during an individual meeting, it may be beneficial for meeting chairs to more deliberately manage the rational and emotional regulation mechanisms, especially for newcomers. Minimising the anxiety and stress on new team members is recommended, in order to give them time to adapt to the procedures and rules of the project meeting. Furthermore, meeting chairs may need to actively manage the negative emotional responses of team members, which they might do by being sensitive to the signals thereof and finding ways to bring affected members back to more constructive roles. 

At the micro-level, the implications are that individual team members should seek to reduce miscommunication, e.g., through careful use of common terminology, and the deliberate establishment of that lexicon for new team members.

The project management standards, such as the PMBOK [6], adopt a closed loop model of managerial processes for planning, executing and controlling processes. The present work suggests that it is necessary for the project manager to take a more adaptive role to the management of human resources. 

Members of the project team have technical roles based on their discipline-specific knowledge and skills—this is well recognised in project management theory. What is less recognised is that each of those team members also has a team role, as shown in Table 1 from [51], which represents their style of communication behavior. Project managers need to manage these roles too. 

From a project management perspective, the results of this study show that the team communication effects that most adversely affect project success are the silent role changes towards disengagement that occur from emotional regulation. It cannot be assumed that team members’ responses will always be rational. Therefore, managing the style of communications within the team (the way members treat each other) and the team roles that people adopt (or are forced into) become a key part of the project manager’s responsibility.

Our suggestions for the further development of the project management body of knowledge are that the standards are more specific about differentiating between technical and team roles. The current guidance on team roles is vague, and may be difficult for practitioners to operationalize. Suggestions for improvements are to (a) describe the types of team roles—we used the taxonomy of [51], but there are others—and to (b) identify the factors that contribute to adverse role changes, especially those factors over which the project manager may exert some control. We consider the sender–receiver model of communication deficit to be useful for representing misunderstanding, but ultimately, it may be too limited for operational use. It fails to represent the large emotional component of peoples’ behavior, and the resulting changes to role adoption. 

### 5.4. Limitations and Future Research Questions

The study had both cross-sectional and longitudinal elements to the data collection, it used both qualitative and quantitative methods, and it compared student engineers to commercially employed engineers. The number of participants in the study was reasonable compared to existing practices in the literature. However, we do point out that the discipline under examination was mechanical engineering, and the team members all had that background. We cannot be sure of the validity of the findings to the many other disciplines of engineering. It would be particularly interesting to examine multidisciplinary engineering projects, since we anticipate that the inhomogeneity of team technical skills may introduce new factors. 

Our study only observed regular official team meetings. Participants communicated with each other on project issues at other times, but this was not examined. 

It could be interesting to explore any correlations between personality (e.g., the five-factor personality model) and team role adoption and change. A quantitative study design, with a large sample size and statistical analysis (e.g., using ANOVA) could be appropriate. Factors of gender and age were not considered in our study because of the limited sample size, but could perhaps be considered alongside a personality study. 

A more qualitative question for future study relates to the communication adjustment trigger; what starts a process of conversational grounding and behavioural changes? At each communication level, the triggers are expected to be different. For example, at the micro-level, this may be a sign of misunderstanding.

We note that the behaviour of participants, especially the managers and supervisors of project teams, were greatly influenced by their job duties, and therefore it was not always easy to distinguish personal preferences in communication (informal team roles) and their expected official behaviour. Consequently, our results should be interpreted primarily for roles adopted by team members, rather than managers. A possible avenue for future research is to examine how social status affects the team roles of subordinates. According to our observations, clients and supervisors had more influence on other participants’ behavioural changes, and hence their role-adoptions.

## 6. Conclusions

This paper identified the changing of communication behaviour and informal team roles that people adopt in engineering projects, and how the adjustment process operates. Since people are key to successful project management, this provides a deeper and more contextualized understanding of the resource management aspect of project management. In particular, this work moves the field forward from a relatively simplistic sender–receiver model of communication. Instead, team members are observed to make constant adjustments of communication style and team roles. Project communication is not just a mechanical process of information exchange; it also includes interpretations of messages (grounding), emotional and rational regulation, and the changing of behaviour patterns (team roles) throughout the project. 

It was observed that participants of engineering project meetings adjusted their communication style to the behaviour of other people and to different communication settings. We suggest that this occurs within three different dynamics: the micro-level (grounding processes in conversation), mezzo-level (emotional and rational regulation) and macro-level (over a period of time). Misunderstanding is attributed to the partial adjustment at one of the levels. Factors predefining communication behaviour change at the macro-level were identified, and a model of communication adjustment at the mezzo-level and at the micro-level was presented.

## Figures and Tables

**Table 1:** Team roles per Nestsiarovich and Pons. Reproduced from [51] with permission.

*N*	Team Roles	Typical Communication Pattern
1	Initiator (initiate process)	Active participation proposes new ideas and tasks, as well as new directions of work.
2	Passive collector (collect information)	Passive data collecting, non-verbal signs of agreement or just short yes/no answer, low verbal participation in team discussion, attentive listening, and keeping ideas inside (non-vocalisation).
3	Explorer (ask questions)	High verbal participation, active data collecting: ask general questions, ask for different facts, ideas or opinions, and explore facts. Ask to clarify or specify ideas, define the term, and give an example.
4	Information provider (provide information)	Provide detailed and excessive information: take an active part in the conversation, but mostly talk rather than listen. This role can be active or passive (Active IP or Passive IP)
5	Facilitator (summarise, control discussion)	Define the task or group problem, suggest a method or process for accomplishing the task, provide a structure for the meeting, control the discussion processes. Bring together related ideas, restate suggestions after the group has discussed them, offer a decision or conclusion for the group to accept or reject. Get the group back to the track.
6	Arbitrator (solve disagreement)	Encourage the group to find agreement whenever a miscommunication arises, or group cannot come to a common position.
7	Representative (express, answer)	Verbalise group’s feelings, hidden problems, questions or ideas that others were afraid to express, provide an answer to questions that were referred to the whole group.
8	Gatekeeper (fill gaps, sensitive to others)	Help to keep communication channels open: fill gaps in conversation, ask a person for his/her opinion, be sensitive to the non-verbal signals indicating that people want to participate.
9	Connector (connect people)	Connect the team with people outside the group.
10	Outsider (stay outside)	Do not participate in project discussion.

**Table 2:** Communication adjustment factors for student engineers, academic supervisors and engineers in commercial organisations.

Factors Affecting Changes in Communication Behaviour	Student Engineers	Academic Supervisors	Engineers in Commercial Organisations
Adjustment to audience	Presence of supervisor or client/boss.		‘Meetings with the software teams are different form the meetings that includes management’. Official style of meetings or presentation was associate with more official behaviour than at team-only meetings where engineers feel more freedom. Furthermore, different teams were observed to have different styles of communication.
Adopted/assigned roles	Chairing a meeting		Engineers were more active in communication when they had much to say about the problem. ‘I am an Initiator and Information Provider, when I am hosting a design proposal meeting for the work I am doing’
Solving progression issues	Feeling that the group or an individual needed their active contribution (‘At some meetings where there was a talking point that was getting stuck I tried to shift the conversation’, ‘When one of our team members was away, I filled the role of Information Provider’).	Changes in their communication behaviour at meetings depended on students’ project progress or client needs. For example, ‘I became more assertive half way through when the client had expressed a concern regarding team achieving goals’, ‘I changed communication style when there were unsolved problems or slow progress in the team’.	‘In some meetings I am the prime driver, in others I am a low-level participant’.
Engagement with depth of discourse			This refers to the professional level of communication (‘more professional level of communication is more challenging’). Participants were more talkative when they felt confidence in the area of discussion: ‘If I am the expert, I will do more information providing’.
Type of meeting			Engineers felt that their communication behaviour depended greatly on the specific details of the particular situation such as the status and quantity of team members in that situation (‘many people give less chance and desire to talk’), the type of meeting. ‘‘Stand-up’ tends to be providing progress updates versus high level design which is more of a how should we do the meeting’.
Defensive behaviour	Less personal progress in project tasks, unprepared meetings, or relatively unknown topic lead to low desire to contribute in discussion (three students). Sensitivity to negative critique, and hence intended to be passive.		
Growth in personal confidence	‘Throughout the year I gained more confidence in the work I had completed’.		
Mood responses	When participants felt tired, unwell, or were just in a bad mood, they were less likely to be active.		

**Table 3:** Communication activity changes for students during the different stages of project development.

Team	Communication Changes with Project Development (from Observation Notes)
1	Students were not very active in the beginning of the project. As the project continued, their activity increased.
2	There was a difference in team behaviour in the beginning and at the end of the project study. In the beginning, students communicated more with supervisor than between each other. They asked a lot of questions and reported results. In the second semester, the situation changed: students became more active in team communication. In the interview responses, some students cited that this was due to having greater confidence.
3	According to the observations and interview data, all students in this team initially were very active in communication. Later some students decided that being active was difficult because of the supervisor’s style (supervisor was a centre of communication and preferred to lead the discussion), so they adjusted their communication behaviour and showed less initiative, talked only when supervisor addressed some questions to them or asked a team. When this happened, student became active, trying to say as much as possible in the short period of time prior to the supervisor started talking again and dominating the conversation.
4	High communication activity in this group was stable during the whole academic year.
5	The team initially was very passive and then activated communication towards the end of semester. Students not only reported to supervisor actively, but also tried to talk with each other to solve problems.

**Table 4:** Team role changes in Team 2 over the academic year.

Participant	Interview Questions: ‘What Is Your Intuitive Perception of Your Own Communication Style in This Team?’ ‘Please Tick All Team Roles (Communication Patterns) That You Think Describe Your Typical Communication Behaviour?’	
	Answer in the First Semester	Answer in the Second Semester
2A	Initially subdued, however taking a larger influence in position over the time due to observing lack of direction or drive in team. Passiveness still preferred to an extent. Explorer, Active Information Provider	I think I try to bring the general thoughts and conversation to a more focused point at times. Initiator, Explorer, Gatekeeper
2B	Observe, comment key points, mostly passive. Representative, Explorer, Gatekeeper	Brief and to the point. Representative, Gatekeeper, Active Information Provider, Passive collector (final meetings)
2C	Seems somewhat relaxed but likes clarification and clear answers so can plan. Prefers being active to be well-informed—asks questions, comments, etc. Representative, Explorer, Facilitator, Gatekeeper	Honest and upfront, perhaps asking a lot of questions but not great at clarifying what I am asking. Representative, Explorer, Passive Collector,
2D	I tend to listen silently and talk only when needed. I focus on my work but check with team members frequently. Passive collector, Representative	I like to hear what everyone has to say and only talk when needed. I only take control of the conversation in which I am proficient. Passive collector, Representative, Gatekeeper, Facilitator (elected)
2E	Open, curious, suggestive. Facilitator, Information Provider, Explorer	Open conversation. Try to get to the bottom of things. Facilitator, Explorer, Passive Collector

## Appendix A

### ***Interview Questions***

1 How comfortable did you feel in this team communication? *(please use scale from 0–10)* What did you like? What was wrong?
2 (students only) Please estimate your contribution to the project? *(please use scale from 0–10)*
3 To what extent did you feel that miscommunication occurs in your meetings? *(never, sometimes, most times, always).* What do you think were the typical causes for this?
4 How productive do you think was your team in problem-solving? *(please use scale from 0–10)* According to you, what were the barriers for team productivity and successful problem-solving? And what were the strong aspects of communication in your team?
5 What is your intuitive perception of your own communication style in this team?
6 Please tick all team roles (communication patterns) that you think describe your typical communication behaviour?
  - Initiator (Initiate process)—Active participation, propose new ideas and tasks, new directions of work.
  - Passive collector (Collect information)—Passive data collecting, non-verbal signs of agreement or just short yes/no answer, low verbal participation in team discussion, attentive listening, and keeping ideas inside.
  - Explorer (Ask questions)—High verbal participation, active data collecting: ask general questions, ask for different facts, ideas or opinions, and explore facts. Ask to clarify or specify ideas, define the term, and give an example.
  - Information provider—Provide detailed and excessive information: take an active part in the conversation, but mostly talk than listen.
  - Facilitator (Summarize, control discussion)—Define the task or group problem; suggest a method or process for accomplishing the task; provide a structure for the meeting, control the discussion processes. Bring together related ideas, restate suggestions after the group has discussed them, offer a decision or conclusion for the group to accept or reject. Get the group back to the track
  - Arbitrator (Solve disagreement)—Encourage the group to find agreement whenever miscommunication arises, or group cannot come to the common division.
  - Representative (Express, answer)—Verbalize group’s feelings, hidden problems, questions or ideas that others were afraid to express, provide an answer to the question that referred to all group.
  - Gatekeeper (Fill gaps, sensitive to others)—Help to keep communication channels open, fill gaps in conversation, ask a person for his/her opinion, be sensitive to the non-verbal signals indicating that people want to participate.
  - Connector (Connect)—Connect the team with people outside the group
  - Outsider—Stay in the room but do not participate in project discussion (think about something else)
7 Do you feel that you changed your communication behaviour at different meetings? Which communication situations caused that?
8 To what extent do you feel that other people’s discussions prevented you from making a contribution at meetings?
9 (students only) If you happened to be elected Team Leader at some meeting, did you feel comfortable in this role? If not, why not?
10 (students only) Do you feel more comfortable at meetings to address your ideas to other students rather than to supervisor and client? Why is that?
11 If you need to say something, which situation is more natural for you: to talk with a particular person or to transmit ideas to the whole team?
12 What is your preferable style of communication at meeting: slow but accurate discussion, middle intensity of communication, or communication at high speed with quick exchanging of ideas? According to you, which meeting style is the most helpful in problem-solving? Which style of communication ‘students-supervisor’ at project discussions do you prefer? (extensive freedom, less freedom, total control). Do you think it predefines the results of project performance? Why?
13 Did you feel that location of the meeting and your position inside the room predefines your communication style? What position was the most comfortable for you?
14 Sometimes people in meetings make use of physical objects like drawings, papers, computer screens, physical models, whiteboard drawing, etc. To what extent did you find it helpful when people presented these types of objects? (never, sometimes, most times, always). Why do you think so? Are there situations where these objects were distracting or caused miscommunication?
